# Units of plasticity in bacterial genomes: new insight from the comparative genomics of two bacteria interacting with invertebrates, *Photorhabdus *and *Xenorhabdus*

**DOI:** 10.1186/1471-2164-11-568

**Published:** 2010-10-15

**Authors:** Jean-Claude Ogier, Alexandra Calteau, Steve Forst, Heidi Goodrich-Blair, David Roche, Zoé Rouy, Garret Suen, Robert Zumbihl, Alain Givaudan, Patrick Tailliez, Claudine Médigue, Sophie Gaudriault

**Affiliations:** 1 INRA, UMR 1133, Laboratoire EMIP, Place Eugène Bataillon, F-34095 Montpellier, France; 2 Université Montpellier 2, UMR 1133, Laboratoire EMIP, Place Eugène Bataillon, F-34095 Montpellier, France; 3 CEA, Genoscope & CNRS-UMR 8030, Laboratoire d'Analyse Bioinformatique en Génomique et Métabolisme, Evry cedex F-91006, France; 4 Department of Biological Sciences, University of Wisconsin, Milwaukee, WI 53201, USA; 5 Department of Bacteriology, University of Wisconsin, Madison, WI 53706 USA; 6 Great Lakes Bioenergy Research Center, University of Wisconsin, Madison, WI 53706 USA

## Abstract

**Background:**

Flexible genomes facilitate bacterial evolution and are classically organized into polymorphic strain-specific segments called regions of genomic plasticity (RGPs). Using a new web tool, *RGPFinder*, we investigated plasticity units in bacterial genomes, by exhaustive description of the RGPs in two *Photorhabdus *and two *Xenorhabdus *strains, belonging to the Enterobacteriaceae and interacting with invertebrates (insects and nematodes).

**Results:**

RGPs account for about 60% of the genome in each of the four genomes studied. We classified RGPs into genomic islands (GIs), prophages and two new classes of RGP without the features of classical mobile genetic elements (MGEs) but harboring genes encoding enzymes catalyzing DNA recombination (RGP_mob_), or with no remarkable feature (RGP_none_). These new classes accounted for most of the RGPs and are probably hypervariable regions, ancient MGEs with degraded mobilization machinery or non canonical MGEs for which the mobility mechanism has yet to be described. We provide evidence that not only the GIs and the prophages, but also RGP_mob _and RGP_none_, have a mosaic structure consisting of modules. A module is a block of genes, 0.5 to 60 kb in length, displaying a conserved genomic organization among the different Enterobacteriaceae. Modules are functional units involved in host/environment interactions (22-31%), metabolism (22-27%), intracellular or intercellular DNA mobility (13-30%), drug resistance (4-5%) and antibiotic synthesis (3-6%). Finally, *in silico *comparisons and PCR multiplex analysis indicated that these modules served as plasticity units within the bacterial genome during genome speciation and as deletion units in clonal variants of *Photorhabdus*.

**Conclusions:**

This led us to consider the modules, rather than the entire RGP, as the true unit of plasticity in bacterial genomes, during both short-term and long-term genome evolution.

## Background

The portion of the bacterial genome common to all strains in a defined set of species and required for basic cellular functions is known as the core genome. The genes variably present between individual strains constitute the flexible genome [1-3]. The estimate of the core and the flexible genomes not only depend on the phylogenetic depth of the group considered, the number of genomes available for comparison but also on the methodology used [3]. Some genes of the flexible genome may play a role in adaptation to special growth conditions, such as those involved in the colonization of new ecological niches, symbiosis, host-cell interaction, and pathogenicity [1,2]. The plasticity of the flexible genome contributes to bacterial genome evolution [2-4].

The flexible genome is organized principally into polymorphic strain-specific DNA segments that are missing in at least one of the genomes analyzed. These segments are named regions of genomic plasticity (RGPs) without any assumption about the evolutionary origin or genetic basis of these variable chromosomal segments [5]. This terminology covers two classes: hypervariable segments that are likely to be the result of deletions of particular DNA regions in one or more strains, and the mobile genetic elements (MGEs).

The plasticity of MGEs depends on three kinds of molecular events, the duplications, inversions and deletions, mediated by transposases and site-specific recombinases whose genes are located either on core genome or on the RGPs themselves [4,6]. The MGEs may be excised from one location and reintegrated elsewhere in the genome or may undergo replicative transposition before integration of a new copy of the element elsewhere in the genome (intracellular mobility). Finally, some MGEs may undergo horizontal genetic transfer (HGT) by natural transformation, transduction or well developed and efficient conjugation mechanisms (intercellular mobility) [4].

The MGE class covers some well characterized elements. Plasmids are stable self-replicating MGEs [4]. Some of them may be transferred in other prokaryotic cells by conjugation. Prophages, the integrated form of temperate bacteriophages, are MGEs that undergo intercellular DNA mobility via transduction [4]. Non replicative MGEs are integrated into the host chromosome and encode at least one enzyme involved in their own excision and integration; these MGEs constitute a large, diverse family [7,8]. They are referred to as (i) transposable elements, if they do not undergo HGT, (ii) genomic islands (GI) if they present features of HGT (phage and/or plasmid-derived sequences, transfer genes, integrases, insertion sequences (IS), G+C content and codon usage bias) but do not encode genes involved in transfer, (iii) integrative mobilizable elements when they require "helper" elements for mobilization and (iv) integrative conjugative elements (ICEs) when they encode their own complete mobility machinery, generally a type 4 secretion system (T4SS) [8]. However, it is often difficult to apply this nomenclature, because MGEs are generally described on the basis of *in silico *analysis in large-scale prokaryotic genome sequencing programs. Thus, the effective excision, intracellular or intercellular mobility and subsequent reintegration via site-specific recombination of MGEs have been demonstrated in only a few cases [9-14]. For these reasons, in the course of genomic projects, in the absence of experimental data, the term "GI" is generally used for putative mobilizable MGEs without the organization typical of prophages [1,2,4,15].

MGEs are potent agents of bacterial genome evolution [4,16]. This property results from both the plasticity of MGE and intra-MGE recombination. Indeed, some MGEs are organized into an array of MGE sub-segments, known as modules [7,17,18]. This mosaic organization is the product of the combination of a limited number of constitutive modules [17]: intracellular mobility modules (recombination and replication functions), intercellular mobility modules (transformation, phage propagation and conjugative transfer) and stability modules. The stability modules are responsible for the maintenance of the MGE in the host cell and encode functions such as poison/antidote systems [19] and antibiotic resistance functions [20,21]. Recombination between MGEs has been studied in a few cases. Deletions and tandem accretions of modules generate hybrid MGEs [22-24]. The bacterial *recA *gene or the recombination systems of the MGEs themselves may mediate the generation of hybrid MGEs [25].

We investigated the plasticity of the flexible genome, by addressing three questions: what are the respective roles of MGEs and hypervariable segments within the flexible genome? Are all RGPs, like MGEs, composed of modules? Do all modules undergo accretion? We addressed these questions by studying the flexible genomes of *Photorhabdus *and *Xenorhabdus*. *Photorhabdus *and *Xenorhabdus *are closely related Enterobacteriaceae [26], both of which are appropriate for genomic evolution studies because of their particular lifestyle [27,28]. *Photorhabdus *and *Xenorhabdus *live in monoxenic cultures within the gut of the soil nematodes, *Heterorhabditis *and *Steinernema*, respectively. These nematodes infect insect larvae, releasing the bacteria into the hemolymph of the insect. The nematode and the bacteria kill the insect and convert the cadaver into a source of food for nematode growth and development. After several rounds of reproduction, the bacteria recolonize the nematodes, which then emerge from the insect cadaver into the soil, to search for a new host [29-31]. This lifestyle, including obligatory, cyclic pathogenic and mutualistic interactions with invertebrate hosts, restricts the ecological niches colonized by *Photorhabus *and *Xenorhabdus*. This biological constraint may favor clonality among bacteria and intrachromosomal rearrangements within the genome. Moreover, *Photorhabdus asymbiotica *has been recovered in clinical isolates from human wounds, in both North America and Australia [32,33]. The emergence of pathogenicity in humans is also consistent with a potential for genomic exchange with environmental bacteria.

Genomic plasticity has been studied to different extents in the two genera. Whole-genome analysis has just begun for *Xenorhabdus *[34], whereas full genome sequences have been published for two *Photorhabdus *strains, revealing the presence of a large number of phage remnants, IS, transposases, repeat elements and overrepresented families of paralogs, consistent with a high level of potential plasticity in these genomes [35,36]. These features are indicative of a general process of genome evolution, as repeatedly observed in host-restricted lineages from many phylogenetic groups [37]. One study described genomic deletion and amplification events in *Photorhabdus *clonal variants obtained in laboratory conditions [38]. These genomic changes are cryptic, but are always found within the Enterobacteriaceae flexible genome. Finally, some studies have characterized a few *Photorhabdus *and *Xenorhabdus *RGPs by *in silico *analysis [35,36,39-43] or by microarray hybridization [38,44].

In this study, we carried out an exhaustive description of RGPs in the genomes of three entomopathogenic strains isolated from nematodes: *Xenorhabdus nematophila *ATCC19061 [34], *Xenorhabdus bovienii *SS-2004 [34] and *Photorhabdus luminescens *TT01 [35], and a strain isolated from humans: *Photorhabdus asymbiotica *ATCC43949 [36]. For the identification of hypervariable regions, recent MGEs, ancient MGEs and non canonical MGEs with unknown mobility mechanisms, we used a new Web tool, *RGPFinder*, which identifies both synteny ruptures in the core genome and classical intrinsic and extrinsic MGE features (Roche, D., unpublished data). We then described the fine modular structure of RGPs and showed that (i) each module is a functional unit, (ii) modules have diverse functions, (iii) modules shape the flexible genomes of the various strains studied and, (iv) some modules are deletion units. Overall, our data strongly suggest that modules are the functional integrated systems serving as the real unit of plasticity within RGPs.

## Results and Discussion

### Identification of regions of genomic plasticity (RGPs)

The size of the flexible genome depends on the methodology used, the depth of phylogenetic comparison and the number of genomes compared [3]. Methods based on genomic comparison, detection of composition bias and search of mobility genes are the most performing tools for the flexible genome characterization [45]. Some methods such as *IslandViewer *and *MobileHomeFinder *are dedicated to predict genomic islands (GIs) with high stringency. Our goal is the identification of the regions of genomic plasticity (RGPs), which covers not only GIs but also rearrangement events without any assumption about the evolutionary origin or genetic basis of these variable chromosomal segments. For this reason, we developed a new Web tool, *RGPFinder*, which combines comparison and composition based approaches (Roche *et al*., unpublished data). Furthermore, *RGPFinder *is specifically designed to identify regions absent from at least one genome inside the comparison genome set. We applied this tool on the genomes of *Photorhabdus luminescens *TT01 (Pl), *Photorhabdus asymbiotica *ATCC43949 (Pa), *Xenorhabdus nematophila *ATCC19061 (Xn) and *Xenorhabdus bovienii *SS-2004 (Xb) strains. We compared the results of *IslandViewer *and *RGPFinder *on our four genomes (data not shown). *RGPFinder *carries out a larger description of the flexible genome (more and larger predicted regions) than *IslandViewer*.

**Figure 1:**
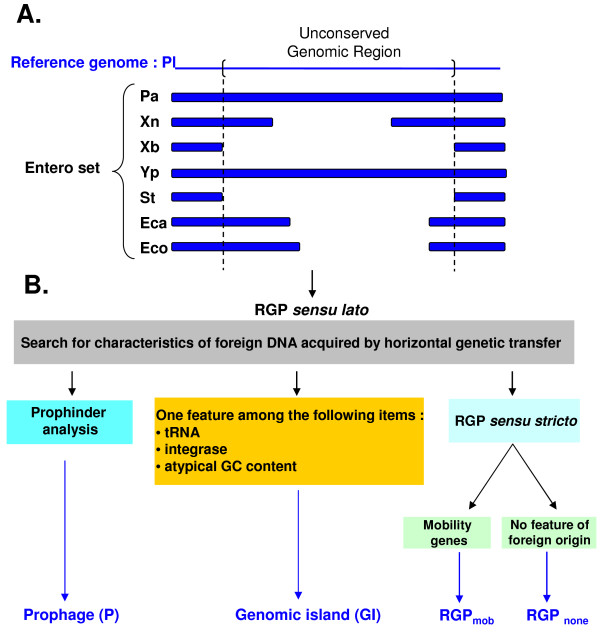
**Strategy for RGP identification and classification in the *Photorhabdus luminescens *TT01 (Pl), *Photorhabdus asymbiotica *ATCC43949 (Pa), *Xenorhabdus nematophila *ATCC19061 (Xn) and *Xenorhabdus bovienii *SS-2004 (Xb) genomes**. **A**. Schematic representation of the procedure used by *RGPFinder *to identify synteny ruptures in the core genome (http://www.genoscope.cns.fr/agc/mage; Roche *et al*., unpublished data). In the example shown, the reference genome, the *P. luminescens *TT01 (Pl) genome, is compared with a bacterial genome set, the Entero set, composed of the *P. asymbiotica *ATCC43949 (Pa), *X. nematophila *ATCC19061 (Xn), *X. bovienii *SS-2004 (Xb), *Yersinia pestis *CO92 (Yp), *Salmonella enterica *subsp. *enterica *Typhi CT18 (St), *Erwinia carotovora *subsp. *atroseptica *SCRI1043 (Eca) and *E. coli *K12 (Eco) genomes. A region of genomic plasticity (RGP) *sensu lato *is the sum of overlapping subregions missing from at least one of the genomes in the bacterial genome set. RGPs have a minimal size of 5 kb. **B**. Classification of the RGPs *sensu lato *as a function of the genetic features identified by *RGPFinder *and *Prophinder *http://aclame.ulb.ac.be/Tools/Prophinder/.

We applied the *RGPFinder *method to different bacterial genome sets (Figure 1.A and Table 1). The first set included strains from the same genus ("Genus" set: Xn *versus *Xb and Pl *versus *Pa). The second set included the four *Photorhabdus *and *Xenorhabdus *strains ("Photo + Xeno" set). The third set, the "Entero" set, included *Photorhabdus *and *Xenorhabus *strains together with other closely related strains from the Enterobacteriaceae (Additional file 1), including the mammalian pathogen *Salmonella enterica *subsp. *enterica *Typhi CT18, the mammalian pathogen *Yersinia pestis *CO92, which also interacts with insects, the plant pathogen, *Erwinia carotovora *subsp. *atroseptica *SCRI1043, and a commensal strain, *Escherichia coli *K12. The proportion of the genome accounted for by RGPs increased with the size of the bacterial genome set: 26-36%, 46-58% and 60-69% for the Genus, Photo + Xeno and Entero sets (Table 1). However, the number of RGPs was similar in the different bacterial genome sets. The nucleus of each RGP was conserved, but was larger when the "Entero" set was used. The characterization of flexible genomes through the comparison of closely related strains can be used to identify RGPs that have recently been acquired or modified, but this technique may result in RGPs generated by more ancient HGT or rearrangements being incorrectly considered part of the core genome[5]. As our objective was the exhaustive characterization of flexible genome plasticity and identification of as many HGT and rearrangement events as possible, we chose to work on the largest comparison set, the Entero set.

**Table 1:** Number and size of regions of genomic plasticity (RGPs) in the *P. luminescens *TT01, *P. asymbiotica *ATCC43949, *X. nematophila *ATCC19061 and *X. bovienii *SS-2004 genomes, according to the set of bacterial genomes used to search for synteny ruptures in the core genome

	Genome	Number of predicted RGPs (% of the whole genome), when compared with the			
	size (bp)	**"Genus" set**^1^*	**"Photo + Xeno" set**^2^*	**"Entero" set**^3^*	"Entero" set after cleaning**
*P. luminescens *TT01	5688987	111 (32.8%)	122 (58.2%)	113 (69%)	**96 (61.5%)**
*P. asymbiotica *ATCC43949	5064808	85 (25.68%)	118 (56.4%)	107 (67.4%)	**92 (59.1%)**
*X. nematophila *ATCC19061	4432590	96 (36.2%)	95 (49.5%)	97 (62.9%)	**83 (54.1%)**
*X. bovienii *SS-2004	4225498	81 (33.1%)	80 (45.8%)	88 (59.6%)	**71 (52.6%)**

* RGPs before manual inspection

** RGPs after manual inspection (see Materials and Methods)

^**1**^*P. luminescens *TT01 and *P. asymbiotica *ATCC 43949 for *Photorhabdus *strains; *X. bovienii *SS-2004 and *X. nematophila *ATCC19061 for *Xenorhabdus *strains.

^**2**^*P. luminescens *TT01, *P. asymbiotica *ATCC 43949, *X. bovienii *SS-2004, *X. nematophila *ATCC19061

^**3**^*P. luminescens *TT01, *P. asymbiotica *ATCC43949, *X. nematophila *ATCC19061, *X. bovienii *SS-2004, *Yersinia pestis *CO92 (accession number NC_003143); *Salmonella enterica subsp. enterica *Typhi (accession number NC_003198), *Erwinia carotovora *SCRI1043 (accession number NC_004547) and *E. coli *K12 (accession number NC_000913).

After manual inspection of the predicted RGPs (see Methods), we obtained a list of 96, 92, 83 and 71 RGPs *sensu lato *for Pl, Pa, Xn and Xb, respectively (Table 1 and see RGPs listed in Additional File 2; these lists are the references used throughout this work.). The RGPs were between 5 kb and 316 kb in length, and more than 50% were less than 20 kb long (Additional File 3). No integral RGP was found to be conserved in all four genomes. The flexible genome of the *Photorhabdus *and *Xenorhabdus *genera accounted for 52.6 to 61.5% of the entire genome (Table 1). In other studies in conditions similar to those used here, the flexible genome has been found to cover: i) 1 to 10% of the genome when serovar or clinical isolates are compared [5,46]; ii) 10 to 40% of the genome when strains are compared [47,48], iii) 25 to 60% of the genome when species are compared [49-51] and iv) 50 to 69% of the genome when genera are compared [52]. Thus, the sizes of the flexible genomes of *Photorhabdus *and *Xenorhabdus *within Enterobacteriaceae were consistent with the findings of other studies.

The flexible genome was found to be larger in *Photorhabdus *than in *Xenorhabdus *genus. This finding is consistent with previous genomic analyses highlighting the importance of genome plasticity in *Photorhabdus *genomes, at both the species [36,40,44] and clonal [38] levels.

### Classification of RGPs

We classified the RGPs into different classes according to their genetic features (Figure 1.B and Table 2). First, we used the *Prophinder *tool [53] to identify prophages (P) (Table 2): Pl, Pa, Xn and Xb have five, eight, six and seven predicted prophages, respectively. P0_PL, P78_PL, P0_PA, P30_PA, P67_XB and P22bis_XN are P2-related phages. This is consistent with previous studies in the *Photorhabdus *and *Xenorhabdus *genera in which P2-phage tail structures were identified [54-58].

**Table 2:** Classification of RGPs in the *P. luminescens *TT01, *P. asymbiotica *ATCC43949, *X. nematophila *ATCC19061 and *X. bovienii *SS-2004 genomes as a function of their genetic composition (the proportion of the modules belonging to a given class is indicated in parentheses)

	Typical MGE		RGP *sensu stricto*	
	
	Prophages	Genomic islands	RGP_mob_^1^	RGP_none_^2^
*P. luminescens *TT01	5 (5%)	38 (39,5%)	23 (24%)	30 (31%)
*P. asymbiotica *ATCC 43949	8 (8,5%)	33 (36%)	10 (11%)	41 (45%)
*X. nematophila *ATCC19061	6 (7%)	32 (38,5%)	27 (32%)	18 (22%)
*X. bovienii *SS-2004	7 (10%)	23 (32%)	21 (30%)	20 (28%)

^1^RGPs that are not prophages, GIs but encode potential DNA recombination enzymes (resolvase, invertase and excisionase)

^2^RGPs that are not prophages, GIs or RGP _mob_

RGPs showing at least one of the typical features of MGEs acquired by HGT (insertion near a tRNA gene, an integrase-coding gene or a G+C content different from that of the core genome) and that are not prophages were named GIs. No ICE class was created since no T4SS loci was identified in the four studied genomes. GI85_PL and GI25_PA, in the Pl and Pa genomes, respectively, were found to harbor type three secretion system (T3SS) loci similar to those of *Yersinia pestis *and *Pseudomonas aeruginosa *[35,36,40,59]. Many pathogenic Gram-negative bacteria encode T3SSs of the Ysc type [60]. In Pl, this T3SS is involved in bacterial adaptation to the insect host, as it prevents the uptake of bacteria by the immunity organs of *Locusta migratoria *[61]. As previously described [30,59], no such loci are found in *Xenorhabdus *genomes. The GI27_PL of Pl is another cluster potentially involved in interactions between bacteria and host, as it harbors a homolog of the *Yersinia *adhesion pathogenicity island (YAPI) [43]. The YAPI encodes a type IV pilus, which contributes to pathogenicity in *Yersinia pseudotuberculosis *serotype O:1, but also includes genes encoding proteins involved in general metabolism, a gene cluster for a restriction-modification system and a large number of mobile genetic elements [62]. This YAPI cluster was detected only on the Pl chromosome. Finally, GI63_XN from Xn is a gene cluster potentially involved in nematode interaction. It harbors the *nil *locus, enabling *X. nematophila *strains to colonize their nematode host, *S. carpocapsae*, specifically [42]. It also encodes putative peptide synthetases, which may be involved in antibiotic production, thereby facilitating the eviction of competing bacteria during the association of nematodes and bacteria before the nematodes leave the insect cadaver. The GI63_XN is specific to Xn.

The other predicted regions were named RGPs *sensu stricto*. Several of these RGPs contained ISs and genes encoding enzymes catalyzing DNA recombination, such as resolvase, invertase and excisionase. We named these RGPs RGP_mob_. RGPs with no remarkable features were named RGP_none_. This last group of RGPs probably consists of hypervariable regions with intracellular mobility mediated by chromosomal rearrangements, such as deletion, duplication or inversion. RGP_mob _and RGP_none _may also be ancient mobilizable MGEs with degraded mobility machinery. The membership to multiple RGP classes of *Photorhabdus *virulence cassettes (PVCs) and the toxin complex (Tc) loci is consistent with this hypothesis. PVCs are phage-like elements flanked by variable putative or identified toxin genes [63-65]). They are found in *Photorhabdus *genomes, but not in *Xenorhabdus *genomes. Yang and coworkers speculated that PVCs might encode phage-like structures, acting as syringes to deliver toxins to the interior of eukaryotic cells [64]. Six of the eight previously described PVC families [36], belong to the prophage and GI classes (PVCcif: GI52_PA, GI60_PL, PVClopT of Pl: GI56_PL, PVCpnf of Pa: GI81_PA, PVClmt of Pa: GI57_PA, PVCphx of Pl and its tandem repeat: P41_PL). The remaining three families were classified as RGP_mob_, despite their putative ancestral origin from phages (PVClopT of Pa: RGP54_PA, PVCphx of PA: RGP66_PA). The Tc loci of *Photorhabdus *and *Xenorhabdus *encoded families of insecticidal toxins active by ingestion [35,36,39,41]. These loci are conserved in several other entomopathogenic bacteria (*Serratia entomophila*, *Pseudomonas entomophila*) and bacteria associated with insects (*Yersinia *spp., *Pseudomonas syringae*) [43,63,66,67], strongly suggesting that HGT of Tc loci has occurred between soil bacteria from different genera known to interact with insects. Some Tc loci are embedded in GIs (GI94_PL, GI27_PL, GI22_PL, GI23_PA, GI91_PA, GI50_XN, GI56_XN, GI23_XB), whereas others are found in RGP_none _(RGP14_PL, RGP58_PL, RGP47_XN, RGP14_XB, RGP30_XB). In these two examples, the genomic erosion of HGT features may lead to a loss of information in some RGPs. This process has classically been reported for nucleotide usage: with increasing time since the HGT event, codon usage in the inserted DNA is gradually modified to match the background of the recipient genome [68]. RGP_mob _and RGP_none _may also be MGE that can be mobilizable *in trans *by other MGEs or non canonical MGEs with mobility mechanisms that have yet to be described. Our classification thus opens up promising new lines of research that may lead to the identification of new classes of MGE.

From the viewpoint of whole-genome evolution, our classification highlights the unusual position of the Pa genome. The proportions of RGP_mob _and RGP_none _are fairly similar in the Pl, Xb and Xn genomes, whereas the Pa genome contains a much higher proportion of RGP_none _(Table 2). This difference probably results from differences in genome evolution and/or plasticity between Pa, which was recovered from a human patient in North America [32], and the other three strains, which were isolated from nematodes. The evolutionary implications of the higher proportion of RGP_none _in Pa remain unclear, but a systematic functional analysis of RGP_none _specific to Pa would complement virulence mapping techniques [29], thereby contributing to the identification of new MGEs involved in the emergence of human pathogens.

### RGPs in the core genome architecture

We mapped the different classes of RGPs on schematic circular maps, to visualize their chromosomal distribution (Additional File 4). GIs, prophages and RGPs *sensu stricto *were found to be evenly distributed throughout the four genomes. Replication, by its inherent asymmetry, shapes the global structure of the prokaryotic chromosome, and some regions of the chromosome are much less accessible for internal recombination than others [15]. However, GI, prophages and RGPs *sensu stricto *were equally likely to be located in the region of the origin of replication, the replication termination region or other regions. This permissiveness probably results from compensatory lateral transfers and/or recombination events, preventing dramatic effects on gene order or the large-scale organization of the genome [15].

We searched for RGP location sites by identifying the genes flanking them on the right and left (Additional File 2). We then looked for RGP location sites conserved within the core genome. We identified 108 hotspots conserved in at least three of the studied genomes, including 14 sites located close to a tRNA gene (tRNA-site) and 94 sites located close to a protein-encoding gene (coding sequence or CDS-site). By definition, tRNA attachment sites are associated with GIs and prophages, whereas coding sequence sites may be associated with all classes of RGPs (Table 3). Therefore, CDS-sites are potential recombination hot spots for hypervariable regions or integration hot spots for MGE or ancient mobilizable MGE. CDS-sites have been little described. However, a recent comparative genomic study in *Escherichiacoli *showed that most gene acquisitions and losses (83%) are adjacent to CDS-sites rather than tRNA-sites [69]. Similarly to tRNA-sites, primary nucleotide sequences (e.g. repeat sequences) or secondary structures close to the CDS-sites may be targeted as integration hot spots. Alternatively, the function of the neighboring gene may be required for intracellular mobility. Indeed, in our study, the genes encoded at CDS-sites were frequently found to be involved in DNA and RNA metabolism or often encoding transferases (Table 3). They may therefore act as cofactors in the excision/integration process.

**Table 3:** RGP sites (genes flanking RGPs on the right or left) conserved in the *P. luminescens *TT01 (Pl), *P. asymbiotica *ATCC43949 (Pa), *X. nematophila *ATCC19061 (Xn) and *X. bovienii *strain SS-2004 (Xb) genomes.

	location sites *	Xn	Xb	Pl	Pa
	*yjeS****_tRNA-gly***	GI10	GI7	GI103	core genome
	*ampH****_tRNA-leu***	GI89	GI10	GI100	P95
	*yjdC****_tRNA-phe***	P75	GI23	GI94	GI23
	*folD****_tRNA-arg***	P75	P22bis	GI95	GI25
	*rpoD****_tRNA-met***	P88	GI29	GI88	P86
	*ylaC****_tRNA-ans***	GI36	GI29	inside GI44	inside GI64
	*ghrA****_tRNA-ser***	GI45	GI33	GI49	GI60
**tRNA gene integration site**	*pgsA_****tRNAleu_tRNAcys***	GI56	P46	GI47	GI61
	*yjeM****_tRNA-pro***	GI69	GI52	GI71	P39
	*mltC***_*tRNAphe***	GI69	GI64	GI27	GI81
	*gltX***_*tRNA-val*_*2tRNAlys*_ *3tRNA*-*val***	GI71bis	GI59	GI33	GI35
	*vacJ***_*tRNA*-*arg***	GI71bis	inside GI55	GI76	inside GI35
	*rsmC***_*tRNA-leu***	GI76	GI67	GI95	GI13
	***yccK_tRNA-ser***	P43	GI16	GI44	GI64

	***aroH***	not present	RGP43	RGP64	RGP48
	***asmA/hisL***	RGP32	core genome	RGP38	RGP68
	***cheZ***	RGP35	RGP30	RGP45	RGP63
	***cpxA/cysE***	GI2	RGP83	GI110	GI104
	***cpxP***	RGP1	RGP82	GI109	P103
	**cspE**	*RGP28bis*	GI61	RGP31	RGP62
	***deaD***	core genome	RGP8	RGP101	RGP96
	***dnaB/zur***	RGP84	P77	RGP96	RGP94
	***dnaJ***	RGP17	RGP27	RGP17	inside RGP15
	***dnaQ***	GI78	core genome	GI26	GI22
	***dxs***	GI22	GI25	core genome	RGP82
	***ecfL***	P88	GI76	GI88	P86
	***eno***	GI20	RGP71	RGP25	inside GI21
**Protein encoding gene integration site**	***exbD***	RGP79	RGP26	RGP87	RGP84
	***fbaA***	RGP65	RGP58	GI27	GI23
	***fis***	GI20	RGP73	RGP92	RGP89
	***flgL***	GP37	G I31	RGP46	GP62
	***flgN***	G I38	GP30	GP45	GP63
	***flhD***	G I36	G I29	G I44	*GI64*
	**fliR**	GI37	RGP48	RGP46	RGP62
	***TfrdA/yhhQ***	RGP15	core genome	RGP93	RGP90
	***gcvP***	RGP25	core genome	GI81	GI27
	***glnG***	RGP95	core genome	RGP5	RGP5
	***glnS***	RGP29	inside RGP22	GI32	RGP73
	***glpD***	RGP96bis	RGP2	GI3bis	GI3
	***gntX (yhgH)***	RGP97	RGP3	RGP4	RGP4
	***gyrB/glmS***	RGP0	RGP0	P0	P0
	***hrpA***	RGP49bis	RGP38	RGP52	GI57
	***kdpE***	RGP29	RGP22	GI33	RGP73
	***leuB***	RGP22bis	GI65	RGP85bis	core genome
	***lysS***	P26	GI64	P80	GI28
	***malM***	GI91	core genome	GI12	GI10
	***mdtA***	core genome	RGP48	GI68	RGP43
	***mdtC/icd***	RGP60	GI49	GI69	GI42
	***mltD***	RGP77	GI57	RGP25	GI21
	***mobB***	GI91	RGP81	core genome	RGP8
	***nagC/ubiG***	core genome	GI60	core genome	GI75
	***nhaA***	GI79	RGP27	RGP17	RGP15
	***nth/yciH***	GI50	core genome	GI56	RGP54
	***pbgE***	RGP40	RGP43	RGP64	RGP48
	***pckA***	RGP100	RGP1	RGP3	RGP3bis
	***pgpA***	RGP19	GI25	core genome	RGP82
	***phoU***	RGP95	RGP4	RGP5	RGP5
	***pntB***	RGP49	GI37	RGP53	inside GI57
	***ppx***	core genome	P32	GI68	RGP43
	***prlC***	RGP96	RGP2	GI3bis	GI3
	***prmB***	RGP71	RGP55	RGP75	GI35
	***pta***	GI63	RGP53	GI73	RGP37
	***purC***	core genome	P46	RGP67	RGP45
	***purK***	core genome	GI59	RGP86	RGP24
	***putP***	GI36	P32	GI47	GI61
	***rpmG***	RGP4	core genome	RGP111	RGP105
	***rpoC***	RGP94	core genome	GI12	GI10
	***rpoH***	RGP16	RGP14	RGP92	RGP89
	***rpoS***	RGP81	core genome	RGP22	RGP19
	***rrmA/guaA***	RGP42	RGP44	RGP66	core genome
	***sbcC***	RGP17	RGP26	RGP87	RGP84
	***smpB***	RGP72	core genome	P80	GI28
	***sprT***	GI22	RGP71	GI85	GI25
	***thiH***	RGP12	RGP79	RGP13 bis	RGP11
	***thrS***	GI39	core genome	RGP65	RGP47
	***tldD***	RGP19	core genome	RGP90	RGP88
	***tpiA***	GI5	RGP82	GI109	P103
	***trmE***	RGP100	P85	RGP112	RGP106
	***typA***	RGP99	RGP5	core genome	RGP6
	***tyrB***	core genome	GI76	GI95	RGP93
	***ung/srmB***	RGP73	core genome	GI79	GI29
	***uvrY***	GI57	RGP47	RGP48	inside GI61
	***valS***	RGP90	RGP9	RGP101	RGP96bis
	***xseA***	P43	RGP45	RGP67	RGP45
	***yafK***	RGP66	GI63	RGP28	RGP79
	***ybhL/folE***	GI31	RGP20	GI37	RGP69
	***yccR***	RGP35	GI16bis	core genome	GI64
	***ychM***	inside GI45	GI33	GI49	GI60
	***yfaE***	RGP65	P15	RGP72	RGP38
	***yfiF***	RGP28bis	GI61	RGP31	core genome
	***ygdE***	RGP86	GI10	core genome	RGP16
	***yheS***	RGP93	core genome	RGP11	GI9
	***yhgF***	RGP96	RGP3	RGP4	inside RGP4
	***yicC***	GI5	P85	RGP112	RGP106
	***yjeP***	core genome	RGP81	RGP104	RGP98
	***ynfK***	core genome	GI37	RGP54	GI57

*location sites are listed when they are conserved in at least three of the genomes panel.

When the two RGP flanking genes are conserved, the gene pair is indicated as location site. If note, each flanking gene is considered as potential location site.

**Figure 2:**
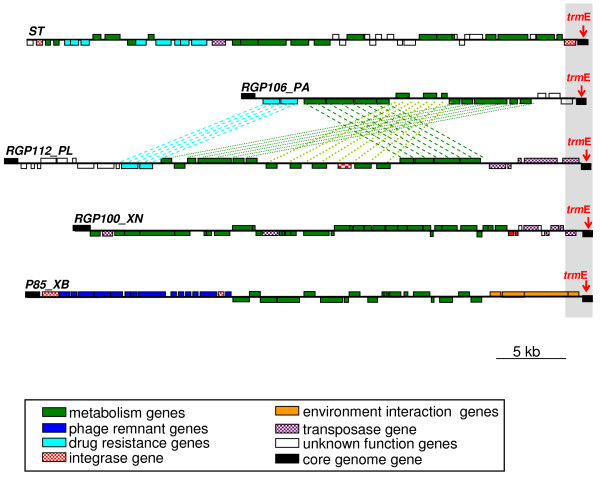
**Example of the *trmE *integration hotspot and its different contents in the *Salmonella enterica typhimurium *(ST), *P. luminescens *TT01 (Pl), *P. asymbiotica *ATCC43949 (Pa), *X. nematophila *ATCC19061 (Xn) and *X. bovienii *SS-2004 (Xb) genomes**. The boxes above and below the axis represent ORFs in the forward and reverse orientations, respectively. The ORF coding for *trm*E is highlighted in gray at the end of each RGP. The putative functions of ORFs within RGPs are indicated by specific colors (see the legend below the figure). Identical subregions are linked by dotted lines.

Very different RGPs were found to be located in the same integration hot spot, in the different *Photorhabdus *and *Xenorhabdus *genomes. For example, we compared the RGPs located within the *trmE *CDS-site (Figure 2). In the *Salmonella enterica *Typhimurium genome, *trmE *is the insertion point for the SG1 genomic island, which includes antibiotic resistance genes and metabolic genes [70,71]. We found no homolog of SGI1 within the *trmE *sites of the *Photorhabdus *and *Xenorhabdus *genomes or elsewhere in the complete genome sequences for these genera. In *Photorhabdus *strains, RGP112_PL and RGP106_PA are located in the *trmE *site. These two RGPs are very similar, differing only in the presence of a large central inversion and additional flanking blocks of genes in Pl. Unlike the *Photorhabdus *genomes, the *Xenorhabdus *genomes had different RGPs at the *trmE *site, RGP100_XN and P85_XB. Some P85_XB gene blocks were found to be located in another region of the *X. nematophila *genome, between the *mrc*A and *cpx*P integration sites (RGP1_XN), highlighting the modular structure of the RGP (see below).

In addition to the *trmE *CDS-site, a number of other integration hotspots are conserved in other bacteria. The CS54 genomic island of *Salmonella enterica *serotype Typhimurium [72] and the 14 kb genomic island of *E. coli *CFT073 [73] are both flanked by the *xseA *integration hotspot. The *rpoS *gene is considered to be a recombination hotspot within the Enterobacteriaceae [74]. Integration hotspots are also conserved in more distantly related bacteria. *flgL *and *fbaA *are the integration sites of the flagellin glycosylation island of *Pseudomonas aeruginosa *[75] and the ICESt1 and ICESt3 of *Streptococcus thermophilus *[76], respectively. The tropism of MGEs for particular integration hotspots in phylogenetically unrelated taxa highlights the probable ancestral role of such sites in integration and recombination.

### Modules are the functional units within RGPs

We have shown that no single RGP is conserved between the four genomes. Indeed, the RGPs identified were either unique or shared a limited number of subregions. An exhaustive *in silico *analysis provided evidence for the structuring of each RGP (P, GI, RGP_mob _and RCP_none_) into several subregions or modules. These modules are blocks of genes 0.5 to 60 kb in length, with a conserved gene order (synteny) in at least two genomes of the Entero set or specific to the strain (see Materials and Methods).

**Figure 3:**
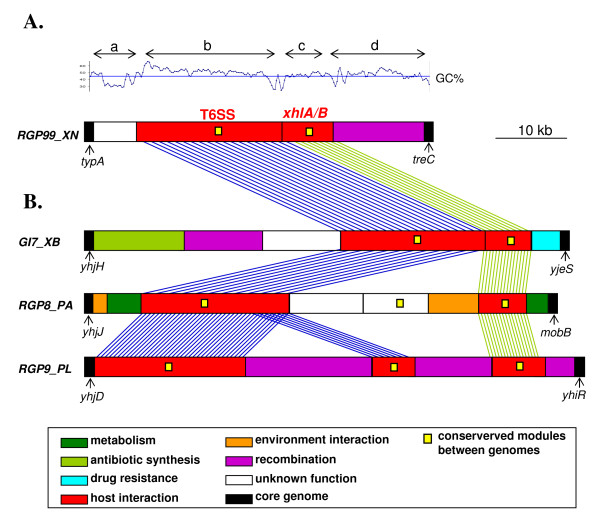
**Schematic diagram of the modular structure of RGP99_XN in the *X. nematophila *ATCC19061 (Xn) genome and of some RGP99_XN module counterparts in the *P. luminescens *TT01 (Pl), *P. asymbiotica *ATCC43949 (Pa), *X. bovienii *SS-2004 (Xb) genomes**. **A**. RGP99_XN can be divided into four subregions or modules, which are distinguished by letters a, b, c, d. G + C is indicated above the modules. **B**. The T6SS (RGP99_XN_b) and XhlA/B (RGP99_XN_c) module counterparts in *P. luminescens *TT01 (RGP9_PL), *P. asymbiotica *ATCC43949 (RGP8_PA) and *X. bovienii *Sj- 2004 (GI7_XB). The boxes represent modules, corresponding to blocks of genes specific to the strain or blocks of syntenic genes, (i.e. with a conserved genomic organization in at least two genomes of the Entero set). The putative biological functions of modules are defined by the specific colors of the box (see the legend below the figure). Yellow squares indicate modules conserved in at least two genomes of the Entero set. Others are modules specific to the strain. The thin vertical black arrows indicate RGP integration sites. The conserved modules in the four genomes are linked by dotted lines. Modules are conserved if they have more than 80% of syntenic genes in common (syntenic genes between two genomes are colocalized genes that shared at least 30% of identity on 80% of the shortest sequence by BlastP). For example, the two modules RGP99_XN_b (T6SS) and RGP99_XN_c (*xhl*AB) are conserved between the four genomes. RGP99_XN_b is composed of 16 genes potentially encoding a type six secretion system (T6SS). The 16 genes display colocalized orthologous genes in XB, PA and PL (identity between orthologs of XN and XB modules: 75% to 97%; identity between orthologs of XN, PL and PA modules: 58% to 91%). RGP99_XN_c is composed of 3 genes, two of them encode an hemolysin belonging to the two partner secretion system family and display colocalized orthologs in XB (73 to 90% of identity), PA (52 to 75% of identity) and PL (51 to 77% of identity).

The modular structure of RGPs *sensu lato *is illustrated by the organization of RGP99_XN in Xn (Figure 3.A). This RGP is located between two genes of the core genome, *typA *and *treC*. RGP99_XN can be broken down into four modules. Two of these modules, RGP99_XN_b and RGP99_XN_c, have a similar organization to gene blocks present in Xb, Pa and Pl. The other two modules, RGP99_XN_a and RGP99_XN_d, are specific to the Xn strain (Figure 3.B). Each module is characterized by a specific G+C content. Furthermore, most of the genes within a given module are involved in similar functions: the RGP99_XN_b module encodes a putative type six secretion system (T6SS; [77]), the RGP99_XN_c module encodes the XhlA hemolysin, which has been implicated in virulence in insects [78], and the RGP99_XN_d module encodes proteins that may be involved in DNA recombination.

For each genome, a list of modules is given in Additional File 5 and the module to which each gene belongs is identified in the gene file accessible on PhotoScope and XenorhabduScope (see Materials and Methods). We distinguished eight functional classes of modules: 1) metabolic modules, consisting of genes involved in primary metabolism, metabolite transport and cell component biosynthesis; 2) drug resistance modules; 3) antibiotic synthesis modules encoding bacteriocins, non ribosomal peptide synthetase, polyketide synthases; 4) phage modules, 5) recombination modules encoding enzymes involved in DNA recombination, such as transposase, invertase, excisionase; 6) environment interaction modules encoding proteins or proteinaceous structures involved in interactions with the environment (iron uptake, adhesion to surfaces etc.), 7) host-interaction modules encoding virulence or symbiosis factors essential for interaction with the insect or nematode host; 8) modules of unknown function.

**Figure 4:**
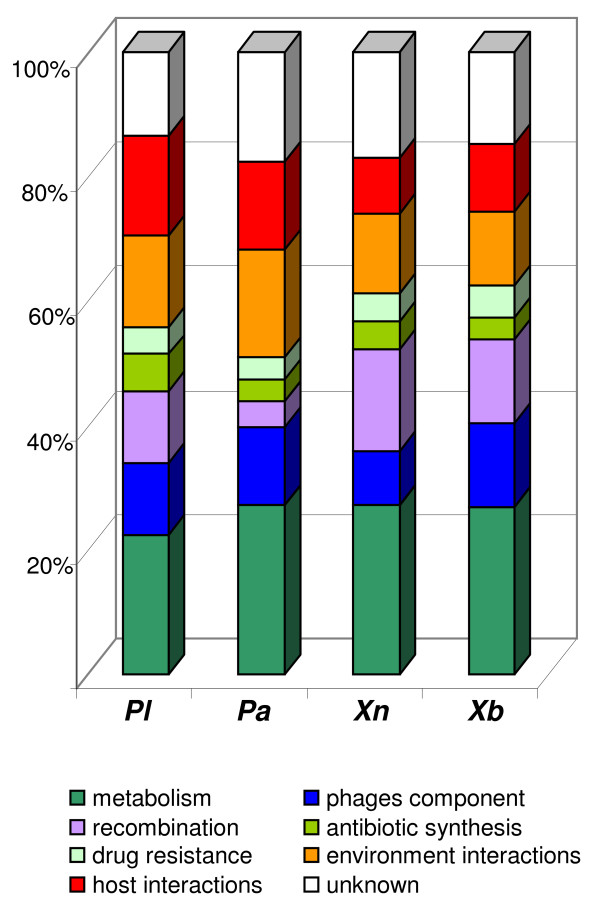
**Module function distribution in the flexible genomes of *Photorhabdus luminescens *TT01 (Pl), *Photorhabdus asymbiotica *ATCC43949 (Pa), *Xenorhabdus nematophila *ATCC19061 (Xn) and *Xenorhabdus bovienii *SS-2004 (Xb). **Eight module functions were defined (see the legend below the figure).

We calculated the proportion of modules belonging to each functional class in the four genomes studied (Figure 4). As previously reported [17], RGPs consist of recombination (4 to 16%) and phage (9 to 14%) modules, each of which mediate intracellular and intercellular DNA mobilization in Eubacteria. Surprisingly, recombination modules were found to be more frequent in the *Xenorhabdus *genomes (13.5% and 16.5%, in Xb and Xn, respectively) than in the *Photorhabdus *genomes (4% and 11% in Pa and Pl, respectively). This difference in recombination module content suggests a difference in recombination strategy between the two genera, with greater recombination activity in the *Xenorhabdus *genomes. This hypothesis is supported by the different patterns of GC skew between the *Photorhabdus *and *Xenorhabdus *genomes. Indeed, whereas the *Photorhabdus *genome GC skews were classical, with two major shifts, one near the origin and the other near the replication termination site, the *Xenorhabdus *genome GC skews were inverted at several points over the chromosome (Additional File 4). These data were confirmed by optical mapping [34] and pulsed-field gel electrophoresis (Ogier and Gaudriault, unpublished data). Such unusual GC skew patterns have already been described in a few genomes [79-82] and were interpreted as the result of recent chromosomal rearrangements, which are commonly observed *in vivo *[79].

Antibiotic modules (3 to 6%) and drug resistance modules (4 to 5%) constitute another canonical module class [17,20]. The *Photorhabdus *and *Xenorhabdus *flexible genomes contain numerous genes and operons encoding antibiotic molecules and secondary metabolites, such as non-ribosomal peptides, polyketides and bacterocins. These products are prime candidates for HGT, because they provide a gain-of-function phenotype and are not essential to the microbial cell [2]. Furthermore, one of the major challenges for entomopathogenic nematode symbiosis is the maintenance of a monoxenic infection in an insect cadaver in the soil. Flexibility, resulting in the renewal of antimicrobial factors, is therefore likely to be determinant for *Photorhabdus *and *Xenorhabdus*.

In addition to the most common building blocks found in MGEs, we identified modules more specifically involved in metabolism, environment/host interaction and unknown functions. Metabolic modules constituted the most frequently represented class, regardless of the strain considered (22-27%). This finding highlights the contribution of the acquisition of additional metabolic traits to adaptability and competitiveness under certain circumstances, such as during the colonization of a new niche or rapidly changing growth conditions [2]. Interactions between host and environment also account for a large proportion of modules (22-31%). This may reflect the complex life style of these bacteria, which interact with two invertebrate hosts, an insect and a nematode. No known function could be attributed to 13 to 18% of the modules, and the hypothetical proteins encoded by the genes of these modules are good candidates for identifying new genes playing a role in the particular lifestyle of these bacteria.

The modular structure of some MGEs has already been reported in previous studies. This structure consists principally of intracellular mobility modules, intercellular mobility modules and antibiotic resistance modules [17,20]. Furthermore, it has been described principally for phages, transposons, ICEs and GIs [24,69,83-89]. By measuring features describing the rate of change of DNA in the locus of enterocyte and effacement of *Escherichia coli*, Castillo and coworkers also concluded that the GI had a mosaic structure and identified subregions, rather than the whole GI, as the true units of selection [90].

In this study, we show that (i) the modular structure relates not only to prophages and GIs but also to RGP_mob _and RGP_none_, (ii) module functions cover a broad range of functions involved in adaptation to the bacterial environment. RGPs *sensu lato *are therefore polyfunctional, and the functional unit of the RGPs is the module. This RGP organization is observed in the *Enterobacteriaceae *family, but probably also throughout the prokaryotic kingdom.

### Modules are the plasticity units of RGPs during long-term genome evolution

As discussed above, no entire RGP is conserved between the four genomes analyzed in this study and RGPs are composed of functional units, the modules. We investigated whether the mobility of these modules within and between cells could have contributed to shaping the RGPs in the different strains or species of a taxon. We compared the distribution of modules throughout the genome and their organization, between the four genomes studied.

**Figure 5:**
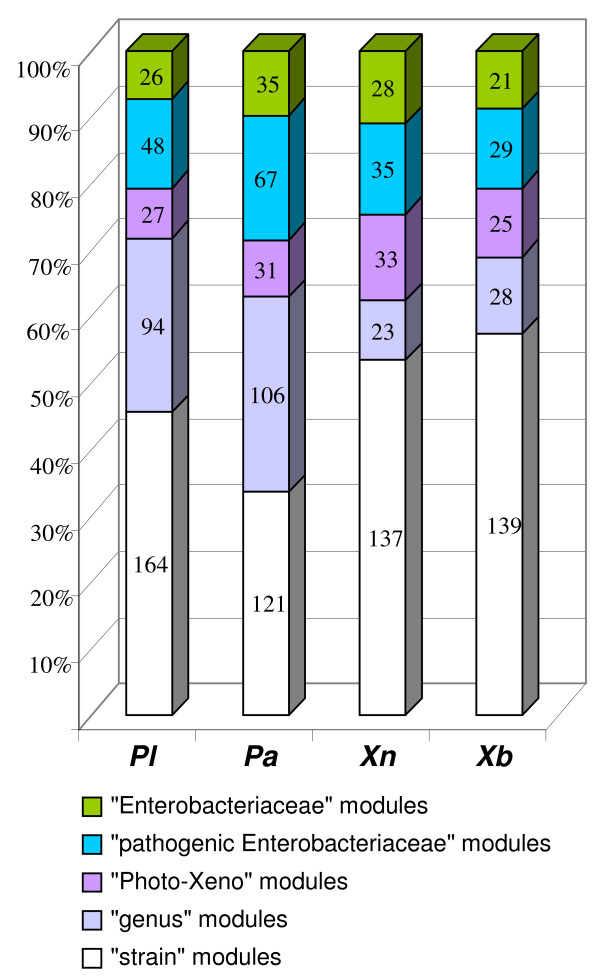
**Distribution of the *P. luminescens *TT01 (Pl), *P. asymbiotica *ATCC43949 (Pa), *X. nematophila *ATCC19061 (Xn) and *X. bovienii *SS-2004 (Xb) modules among five subsets: **i) modules found exclusively in the genome of the strain of interest ("strain" modules); ii) modules found exclusively in the genomes of the two *Photorhabdus *strains or the two *Xenorhabdus *strains ("genus" modules); iii) modules present in at least one *Photorhabdus *genome and one *Xenorhabdus *genome but absent from genomes of other bacteria of the "Entero" set ("Photo-Xeno" modules), iv) modules found in the genomes of pathogenic bacteria of the "Entero" set ("pathogenic Enterobacteriacae" modules) and v) modules found in the non pathogenic *E. coli *K12 strain ("*Enterobacteriaceae*" modules).

We first analyzed the distribution of modules within the Entero set initially used for RGP characterization (Figure 5). We defined five classes of module as a function of their conservation within a taxonomic group: i) modules found exclusively in the genome of the strain of interest ("strain" modules); ii) modules found exclusively in the genomes of the two *Photorhabdus *strains or the two *Xenorhabdus *strains ("genus" modules); iii) modules present in at least one *Photorhabdus *genome and one *Xenorhabdus *genome but absent from the genomes of other bacteria of the "Entero" set ("Photo-Xeno" modules), iv) modules found in genomes of pathogenic bacteria of the "Entero" set ("pathogens" modules) and v) modules found in the non pathogenic bacterium *E. coli *K12 strain ("Enterobacteriaceae" modules). The modules belonging to "Photo-Xeno", "Enterobacteriaceae" and "pathogens" groups were evenly distributed in the four strains, together accounting for between 30 and 40% of the modules (Figure 5). There were relatively few "Photo-Xeno"-specific modules (7% to 13%). Therefore, despite having very similar lifestyles (entomopathogenic bacteria living in symbiosis with nematodes) and being closely related phylogenetically, the flexible genomes of *Xenorhabdus *and *Photorhabdus *were different, suggesting different mechanisms of molecular adaptation to the environment and hosts of these two genera. Most of the modules in these four genomes were of the "strain" and "genus" types, these two types of module together accounting for about two thirds of the modules. However, the *Photorhabdus *genus had similar proportions of "strain" and "genus" modules, whereas the *Xenorhabdus *genus had a higher proportion of "strain" modules (54 to 57%) than of "genus" modules (9% to 11%). Thus, the flexible genome of *Xenorhabdus*, unlike that of *Photorhabdus*, is mostly strain-specific. This may reflect the closer phylogenetic relationship between *P. luminescens *and *P. asymbiotica *than between *X. nematophila *and *X. bovienii *[26].

**Figure 6:**
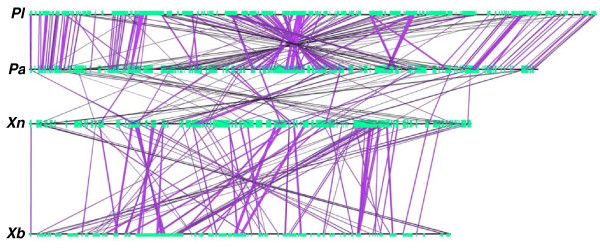
**Schematic representation of the genomic organization (synteny) of "genus" and "Photo-Xeno" modules conserved between *P. luminescens *TT01 (Pl), *P. asymbiotica *ATCC43949 (Pa), *X. nematophila *ATCC19061 (Xn) and *X. bovienii *SS-2004 (Xb).** Each genome is represented by a horizontal line, with the RGPs *sensu lato *represented as green boxes. Purple lines indicate the conservation of modules between two adjacent chromosomes(the representation is dependent on chromosome order on the figure, *i.e*. a module conserved between *X. bovienii *and P*. luminescens *will not be drawn).

We then assessed module synteny between the four strains studied, by aligning conserved modules on linear genomic maps (Figure 6). Studied strains share little module synteny with each other, particularly *Xenorhabdus *species. As an illustration of this absence of synteny between modules, we further analyzed the genomic organization of modules initially described in RGP99_XN, in the four genomes studied (Figure 3). In the Xn, Pl, Pa and Xb genomes, the T6SS and XhlA/B modules were both found in a single RGP but they are not located in the same integration site, as these modules were flanked by different genes of the core genome. The T6SS and XhlA/B modules are either in a syntenic block (Xn and Xb), intercalated with additional modules (Pa and Pl) or partially duplicated (Pl). They are also flanked by additional modules specific to the strain or conserved inside others RGPs. A patchy structure has already been described for MGEs, consistent with horizontal transfer leading to the gradual stepwise construction of these MGEs [69,90-92].

Our data show that the RGPs of the flexible genome are shaped by the acquisition and loss of modules, and that RGP diversity probably results from intrachromosomal or interchromosomal rearrangements between module units.

### Modules are the units of deletion of RGPs involved in short-term genome rearrangements

If modules do indeed shape the RGPs during genome evolution, some rearrangements are likely to be detectable in clonal populations during growth in the laboratory. We therefore searched for intrachromosomal rearrangements of modules within the genome of Pl clonal variants available in our laboratory. TT01α is a Pl variant collected from a laboratory-maintained symbiotic nematode [38]. TT01α differs from the Pl reference genome by nine large-scale deletion events in the flexible genome, but these genomic changes have cryptic phenotypic consequences. The boundaries of the deleted regions in TT01α were located in the Pl genome by a combination of macrorestriction and DNA microarray experiments [38].

**Figure 7:**
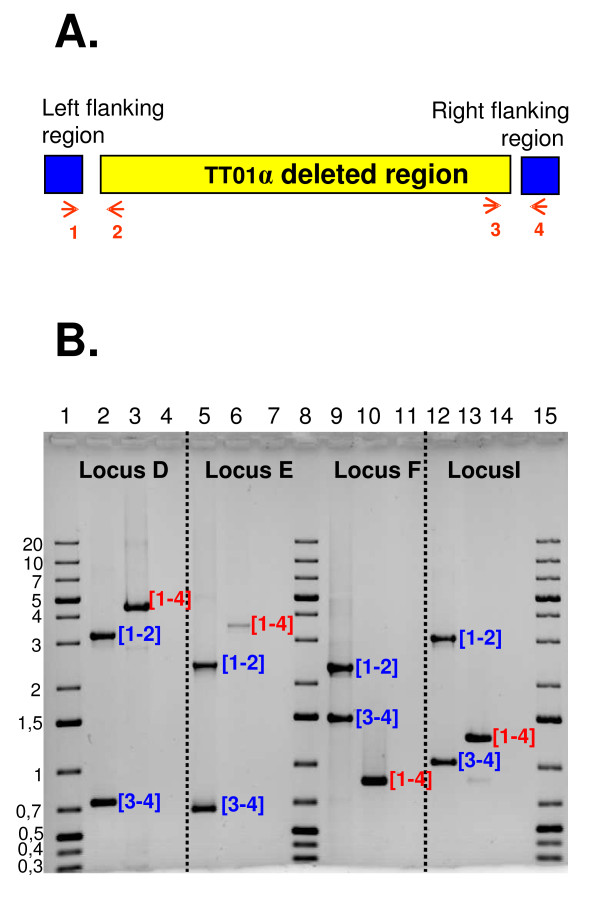
**Boundaries of deleted regions in the genome of the *P. luminescens *TT01α variant match with the module boundaries defined in the *P. luminescens *TT01 (Pl) genome**. A. Schematic diagram of the strategy used for multiplex PCR amplification. The yellow box indicates the deleted region. Blue boxes represent the flanking regions. Red horizontal arrows indicate the location of the primers. The primer mixture (P1, P2, P3 and P4) was designed to amplify two fragments from the Pl genome and only one fragment if the region is effectively deleted, as predicted for the *P. luminescens *TT01α variant genome. B. Agarose gel electrophoresis of the PCR products generated by amplifying the genomic DNA of Pl (lanes 2, 5, 9, 12), of the TT01α variant (lanes 3, 6, 10, 13) or of water (lanes 4, 7, 11, 14) with primers P1, P2, P3 and P4, designed as indicated in (A) for loci D (lanes 2-4), E (lanes 5-7), F (lanes 9-11) and I (lanes 12-14). Loci D, E, F and I are four regions of the Pl genome identified as missing in the TT01α variant genome (see text for details). [1-2], [3-4] and [1-4]. indicate bands of sizes compatible with amplification of the regions between the P1 and P2 primers, the P3 and P4 primers and the P1 and P4 primers, respectively. Lanes 1, 8, 15: molecular markers. The sizes of fragments are indicated in kb to the left of the gel.

We first compared the boundaries of the deleted regions and the module boundaries. All the regions deleted in TT01α were found to be embedded in an RGP, and seven of the nine regions matched with one or several modules. We analyzed the precise boundaries of four selected loci within these seven deleted regions: locus D, which matches module RGP45_PL_a and encodes metabolism and drug resistance functions; locus E, which matches module RGP53_PL_c and encodes antibiotic biosynthesis functions; locus F, which matches with modules GI59_PL_a, b and encodes metabolism and host interaction functions; locus I, which matches with modules P80_PL a, b, c, d, e, f and encodes metabolism and phage functions [38]. For each locus, we performed multiplex PCR amplification with two primer pairs flanking the left (primer 1/primer 2) and the right (primer 3/primer 4) boundaries of the regions deleted in TT01α (Figure 7.A). As predicted by macrorestriction and DNA microarray experiments, PCR amplification generated two fragments (fragments [1-2] and [3-4] ) when Pl genomic DNA was used as the template, and one fragment (fragment [1-4]) when TT01α genomic DNA was used as the template (Figure 7.B), confirming the occurrence of a deletion event in the TT01α genome. We mapped the deletion boundaries more precisely, with the goal of obtaining an exact picture of the result of the deletion making it possible to predict the intrachromosomal rearrangements occurring at these loci, by sequencing the four fragments [1-4] obtained from the TT01α genome.

**Figure 8:**
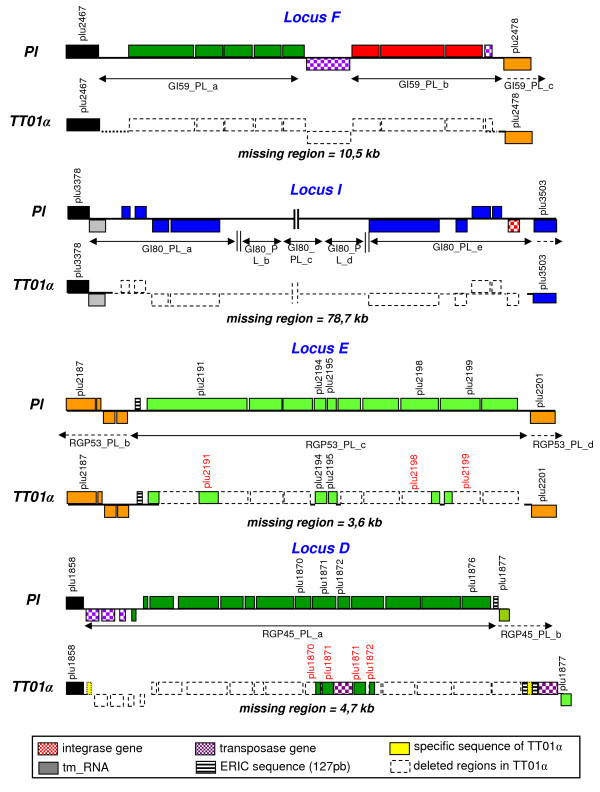
**Schematic genetic map of the F, I, E and D loci in the Pl genome and their counterparts in the TT01α variant genome**. The boxes above and below the axis represent ORFs in the forward and reverse orientations, respectively. The putative functions of the ORFs within RGPs are indicated by specific colors (see legend Figure 4). Mobility genes, repeat sequences or specific features are marked with motifs described below the figure. The modules in loci D, E, F and I are indicated, below the axis, by black horizontal double arrows.

For all four fragments, the sequence data confirmed the predicted deletion boundaries: the D, E, F, I locus boundaries exactly matched the module boundaries identified by *in silico *analysis, validating our modularization procedure. Moreover, we distinguished two classes of deletion patterns potentially matching at least three deletion scenarios (Figure 8). At loci F and I, which are embedded in GIs, the whole module or block of modules was found to be missing in the TT01α genome, suggesting that a single block deletion event led to the Pl/TT01α transition in this part of the genome. In both cases, a gene encoding an enzyme involved in DNA recombination (transposase for locus F and integrase for locus I) is located at the internal border of the locus and is therefore a good candidate for involvement in the rearrangement. By contrast, at loci D and E, which are embedded in RGPs *sensu stricto*, only subregions of modules are missing in the TT01α genome, consistent with the occurrence of more complex rearrangement events in these genomic areas during the Pl/TT01α transition. Locus E in the TT01α genome consists of five fragmented remnants of the initial locus E. Genomic reduction has probably occurred in several stages at locus E, although the absence of mobile elements or genes encoding recombination functions makes it difficult to determine the genomic rearrangement scenario. Locus D in the TT01α genome displays a shuffling pattern at three locations: a 105 bp nucleotide sequence at the left internal border; a region composed of two Enterobacteriaceae repeat intergenic consensus (ERIC) sequences, a transposase-encoding gene and a nucleotidic sequence of 154 bp at the right internal border; a region composed of the truncated *plu1870 *and *plu1872 *genes and the *plu1871 *gene interrupted by a transposase gene in the middle part. As the elements within these shuffled subregions are probably remnants of molecular actors involved in the rearrangements of locus D, we analyzed them further. ERIC elements are miniature (127 bp) non autonomous mobile elements in Enterobacteriaceae genomes [93,94]. The Pl genome is particularly rich in such repeats [35]. The two transposase genes encoded a transposase of the IS928 family. The 105 and 154 bp sequences are palindromic nucleotide sequences consisting of fragments dispersed in the Pl genome. They display no sequence similarity to each other or to ERIC and the two transposase genes. The inserted transposases and ERIC sequences clearly played a role in the plasticity of locus D, but the origin and role of the exogenous 105 bp and 154 bp nucleotide sequences remain unknown.

In conclusion, whatever the molecular mechanism involved in these deletion scenarios, in the case of clonal genomic plasticity, the modules may be deleted over a timescale corresponding to growth in the laboratory and may be considered units of deletion within RGPs.

## Conclusions

The data presented here participate to a better vision of the bacterial flexible genome organization. The characterization of RGPs by the *RGPFinder *method showed the flexible genome to be much broader than the sum of GIs and prophage elements. Additional elements -- RGP_mob _and RGP_none _elements -- lacking classical mobility features may be hypervariable regions that undergo deletions, ancient mobile elements with a degraded mobilization machinery, MGE that can be mobilizable *in trans *by other MGEs or non canonical MGE for which the mobility mechanism has yet to be described. Furthermore, we provide evidence that not only GIs and prophages, but all RGPs *sensu lato *have a mosaic structure composed of modules that are both functional and plasticity units.

The application of comparative genome sequencing to experimental evolution studies provides us with an opportunity to study the link between genome dynamics and adaptive evolution. Nevertheless, such studies are generally carried out on bacterial populations evolving in a synthetic broth culture, and they mostly identify point mutations [95,96]. Here, by carrying out comparative genomics studies on variants obtained from their host in the laboratory, we showed experimentally that the same modules undergo genomic rearrangements during genome speciation and short-term genomic rearrangements. This work improves our understanding of the process responsible for bacterial genome diversification and evolution.

Obtaining of these data were made possible by the use of the *Photorhabdus *and *Xenorhabdus *genera for our comparative genomic study. Indeed, the life cycle of these genera is restricted to two successive ecological niches. We argue that this unusual pattern of selective pressure is responsible for an alternation of genomic shuffling: HGT in the insect cadaver, which constitutes an abundant nutrient resource potentially shared with many other microorganisms and intrachromosomal rearrangements of recently acquired modules in the bacterial monoxenic culture within the nematode gut, as observed in the Pl variant isolated from a laboratory-maintained symbiotic nematode. We therefore suggest that *Photorhabdus *and *Xenorhabdus *are suitable new bacterial models for studies of the evolution of bacterial genomes.

Finally, the data obtained in this study contribute to our understanding of the fluid nature of genomes throughout the kingdoms of life. According to J. A. Shapiro, prokaryotic and eukaryotic cells are genetic engineers and mobile elements are "natural genetic engineering systems" that facilitate the evolutionary rewriting of genomic information [97]. Shapiro's hypothesis is that repeated evolutionary challenges have selected systems that (i) reduce the size of the genomic search space and (ii) maximize the chance of success by using combinatorial processes based on basal functional components [97]. We argue that the modules described here are entirely consistent with this vision, as these functional units recombined at a limited number of hotspots shaping and delimiting the flexible genome.

## Methods

### Bacterial strains and genome sequences

*Photorhabdus luminescens *subspecies *laumondii *strain TT01 is a symbiont of the nematode *Heterorhabditis bacteriophora *isolated in Trinidad and Tobago [35,98]. The genome of strain TT01 consists of a single circular chromosome of 5,688,987 bp (accession number NC_005126). *Photorhabdus asymbiotica *subspecies *asymbiotica *strain ATCC43949 is a North American clinical isolate. This strain was isolated in 1977 from a female patient with endocarditis, in Maryland, USA [32,99]. The genome of strain ATCC43949 consists of a single circular chromosome of 5,064,808 bp and a 29,732 bp plasmid [36] (accession numbers NC_012962 and NC_012961, respectively). *Xenorhabdus nematophila *ATCC19061, the type strain of the species, is a symbiont of the nematode *Steinernema carpocapsae*, isolated from Georgia, USA [100]. The genome of strain ATCC19061 comprises a single circular chromosome of 4,432,590 bp and a 155,327 bp plasmid [34] (accession numbers FN667742 and FN667743, respectively). *Xenorhabdus bovienii *SS-2004 is a symbiont of the nematode *Steinernema jollieti *sp. isolated from a woodland in the Missouri valley, USA, in 1999 [101]. The genome of strain SS-2004 comprises a single circular chromosome of 4,225,498 bp [34] (accession number FN667741). The four genomes were input into the PhotoScope and XenorhabduScope databases http://www.genoscope.cns.fr/agc/mage.

### RGP identification

Regions of genomic plasticity (RGPs) were sought in the *P. luminescens *TT01, *P. asymbiotica *ATCC43949, *X. nematophila *ATCC19061 and *X. bovienii *SS-2004 genomes, with the *RGPfinder *web tool implemented in the MaGe annotation platform (http://www.genoscope.cns.fr/agc/mage; Roche *et al*., unpublished data). Briefly, *RGPFinder *searches for breaks in synteny between a reference genome and the genomes of a set of related bacteria -- the bacterial genome set (Figure 1.A). A RGP *sensu lato *is the sum of overlapping subregions missing in at least one of the bacterial genomes of the comparison set. RGPs have a minimal size of 5 kb. This excludes the isolated insertion sequences of the RGPs, but favors regions with several genes of potential functional interest in the bacterial biology. This definition does not involve any underlying assumption about the evolutionary origin or genetic basis of these variable chromosomal segments. *RGPFinder *also provides information about composition abnormalities (GC% deviation, codon adaptation index) and about the features flanking the RGPs, such as tRNA, IS, integrase (int) and genetic elements involved in DNA mobility (mob), which are common characteristics of foreign DNA acquired by horizontal genetic transfer. The results obtained with this web tool include those for Alien Hunter [102], a method detecting atypical sequences (*i.e*., sequences potentially acquired by horizontal genetic transfer) through the analysis of composition bias.

Predicted RGPs were then manually inspected, to eliminate false-positive results. Indeed, point mutations may lead *RGPFinder *to identify a region as an RGP when it actually belongs to the core genome. Finally, the boundaries of the RGP were homogenized between the compared genomes and potential insertion sites were defined. The genomes used in the bacterial genome set were those of *P. luminescens *TT01, *P. asymbiotica *ATCC43949, *X. nematophila *ATCC19061, *X. bovienii *SS-2004, *Yersinia pestis *CO92 (accession number 003143); *Salmonella enterica *subsp. *enterica *Typhi CT18 (accession number NC_003198), *Erwinia carotovora *subsp. *atroseptica *SCRI1043 (accession number NC_004547) and *E. coli *K12 (accession number NC_000913). Finally, *Prophinder *was used to detect prophages among the RGP *sensu lato *[53], http://aclame.ulb.ac.be/Tools/Prophinder/.

### Definition and distribution of modules

The *MaGe *web interface [103] was used to divide RGPs manually into subregions corresponding to blocks of genes specific to the strain or blocks of syntenic genes (i.e. genes with a conserved genomic organization in at least two genomes of the Entero set). These subregions, which often contain genes of similar biological function, were named "modules". The distribution of modules among the "Enterobacteriaceae" genome set was analyzed manually: a module was considered present (or partially present) in a genome if it had more than 80% (25%) of syntenic orthologous genes (orthologous genes shared at least 30% of identity on 80% of the shortest sequence by BlastP) with the module of the reference genome. The module was otherwise considered to be absent. Descriptions of the modules and their distributions are available from PhotoScope https://www.genoscope.cns.fr/agc/mage/wwwpkgdb/Login/log.php?pid=13 and XenorhabduScope https://www.genoscope.cns.fr/agc/mage/wwwpkgdb/Login/log.php?pid=24, by opening the Genomic Object Editor of a gene and consulting the "Module" results.

### Multiplex PCR procedure and sequencing

Genomic DNA was extracted as previously described [44] and stored at 4°C. Primers flanking the right (primers P1/P2) and left (primers P3/P4) module borders were designed with Primer 3 http://frodo.wi.mit.edu/primer3/. Primer sequences are listed in Additional File 6. Multiplex PCR with the four primers (P1, P2, P3, P4) was performed with a Bio-Rad thermocycler (Bio-Rad, Marne La Vallée, France). Fragments with a predicted size smaller than 3 kb were amplified with Invitrogen *Taq *polymerase (Invitrogen, France), according to the manufacturer's protocol. Fragments with a predicted size greater than 3 kb were amplified with the Herculase Enhanced DNA polymerase (Stratagene, Amsterdam Zuidoost, Pays Bas) in accordance with the manufacturer's recommendations. Samples of reaction mixtures were analyzed by electrophoresis in an agarose gel. The fragments amplified by PCR [P3-P4] were purified from the gel with the high purity purification kit from Roche (Roche Diagnostic, France) and sequenced with the PCR primers described in Additional File 6, via a chromosome walking process, by Macrogen (South Korea).

## List of abbreviations

Eco: *Escherichia coli *K12; Eca: *Erwinia carotovora *subsp. *atroseptica *SCRI1043; GI: genomic island; HGT: horizontal genetic transfer; ICE: integrative conjugative element; int: integrase; IS: insertion sequence; MGE: mobile genetic element; P: prophage; Pa:, *Photorhabdus asymbiotica *ATCC43949; Pl: *Photorhabdus luminescens *TT01; PVC: *Photorhabdus *virulence cassette; RGP : region of genomic plasticity; St : *Salmonella enterica *subsp. *enterica *Typhi CT18; T3SS : type three secretion system; T6SS: type six secretion system; Tc: toxin complex; Xn: *Xenorhabdus nematophila *ATCC19061; Xb: *Xenorhabdus bovienii *SS-2004; YAPI: *Yersinia *adhesion pathogenicity island; Yp: *Yersinia pestis *CO92

## Authors' contributions

J-CO, AG, CM, PT, SG designed the project. SF, HG-B, GS provided genomic data. JCO carried out *in silico *description of the RGPs, their modularization and the PCR experiments. AC, DR, ZR contributed in computational analysis. J-CO, AC, RZ, AG, PT, CM, SG analyzed the data. J-CO and SG wrote the paper with contributions from AG. All authors read and approved the final manuscript.

## Supplementary Material

Additional File 1 **Phylogenetic tree for the *Enterobacteriaceae *derived from a distance analysis of 16S rRNA gene sequences**. The genomes used in this study belong to species indicated in red (*Photorhabdus *and *Xenorhabdus*) and blue (other Enterobacteriaceae). *Vibrio cholerae *(Vibrionaceae) was used as an outgroup. The GenBank accession numbers of the sequences are shown in brackets. Bootstrap values of more than 50% are indicated at the nodes. The bar indicates 1% sequence divergence. A figure showing a phylogenetic tree for the Enterobacteriaceae used in this study. Click here for file

Additional File 2 **List of regions of genomic plasticity (RGPs) in the *P. luminescens *TT01 (Pl), *P. asymbiotica *ATCC43949 (Pa), *X. nematophila *ATCC19061 (Xn) and *X. bovienii *SS- 2004 (Xb) genomes**. A table listing the RGPs. Click here for file

Additional File 3 **Distribution of RGP sizes in the *Photorhabdus luminescens *TT01 (Pl), *Photorhabdus asymbiotica *ATCC43949 (Pa), *Xenorhabdus nematophila *ATCC19061 (Xn) and *Xenorhabdus bovienii *SS-2004 genomes (Xb)**. A figure showing the distribution of RGP size. Click here for file

Additional File 4 **Schematic diagram of the distribution of RGPs *sensu lato *on the circular chromosomes of *P. luminescens *TT01 (Pl), *P. asymbiotica *ATCC43949 (Pa), *X. nematophila *ATCC19061 (Xn) and *X. bovienii *SS-2004 (Xb)**. Successive circles from inside to outside: GC skew; GC deviation (with values exceeding +/- 2 standard deviations indicated in red). Distribution of the different RGP types: GIs (orange), Phages (green) and RGP_mob _and RGP_none _(yellow). A figure showing schematic diagrams of the distribution of RGPs. Click here for file

Additional File 5 **List of modules in the *P. luminescens *TT01 (Pl), *P. asymbiotica *ATCC43949 (Pa), *X. nematophila *ATCC19061 (Xn) and *X. bovienii *SS-2004 (Xb) genomes and their distribution in the *Yersinia pestis *CO92 (Yp), *Salmonella enterica *subsp. *enterica *Typhi CT18 (St), *Erwinia carotovora *subsp. *atroseptica *SCRI1043 (Eca) and *E. coli *K12 (Eco) genomes**. A table listing the modules. Click here for file

Additional File 6 **Primers used in the study**. A table listing the primers used in this study. Click here for file
