# Supplementary figures and images for: Units of plasticity in bacterial genomes: new insight from the comparative genomics of two bacteria interacting with invertebrates, Photorhabdus and Xenorhabdus
Source: BMC Genomics. 2010 Oct 15;11:568. doi: 10.1186/1471-2164-11-568 (PMC3091717; doi:10.1186/1471-2164-11-568)

Additional file 1

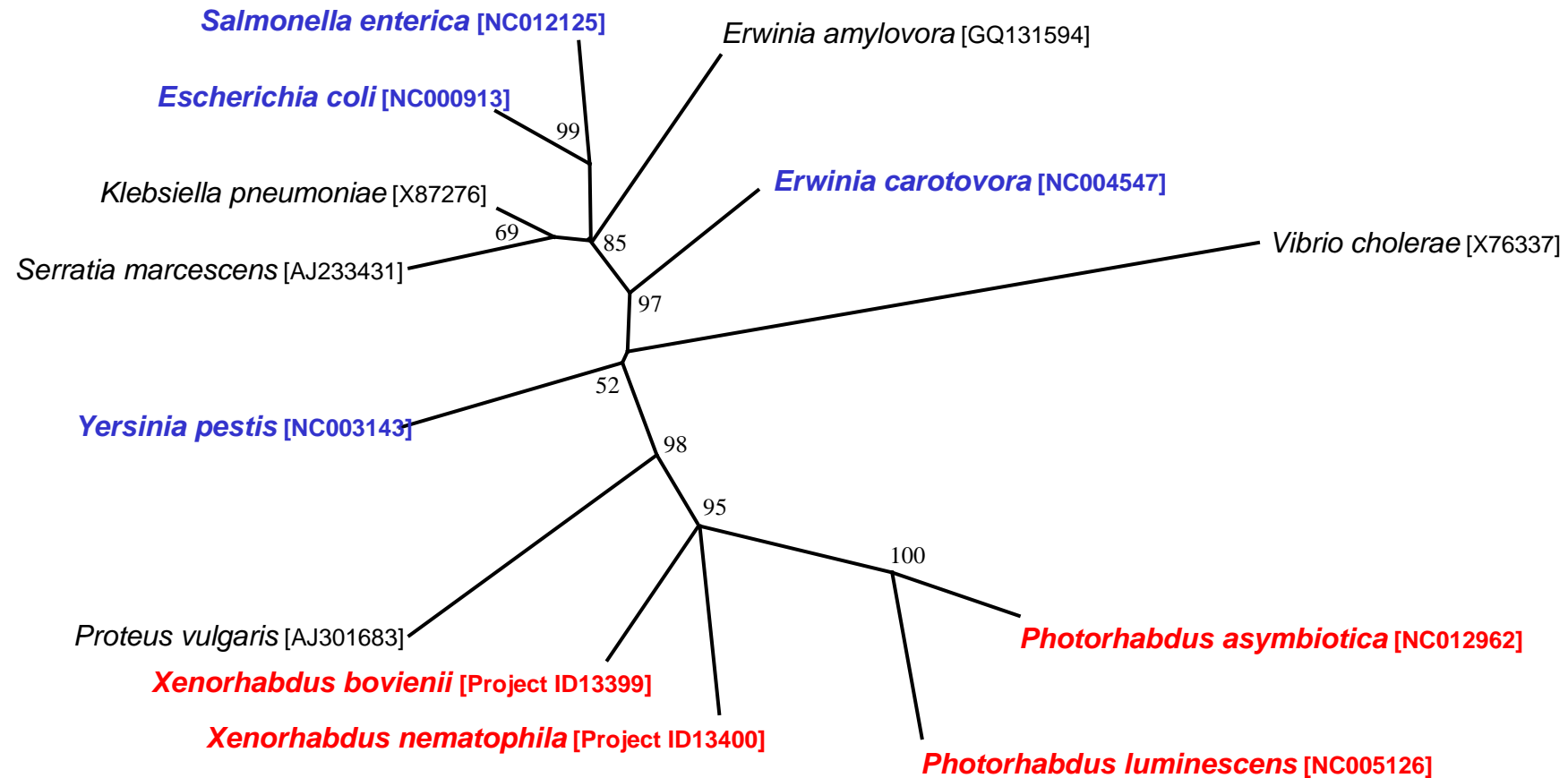

0.01

Supplement: Additional File 1 — Phylogenetic tree for the Enterobacteriaceae derived from a distance analysis of 16S rRNA gene sequences. The genomes used in this study belong to species indicated in red (Photorhabdus and Xenorhabdus) and blue (other Enterobacteriaceae). Vibrio cholerae (Vibrionaceae) was used as an outgroup. The GenBank accession numbers of the sequences are shown in brackets. Bootstrap values of more than 50% are indicated at the nodes. The bar indicates 1% sequence divergence. A figure showing a phylogenetic tree for the Enterobacteriaceae used in this study. [file 1471-2164-11-568-S1.PDF]

Additional File 3

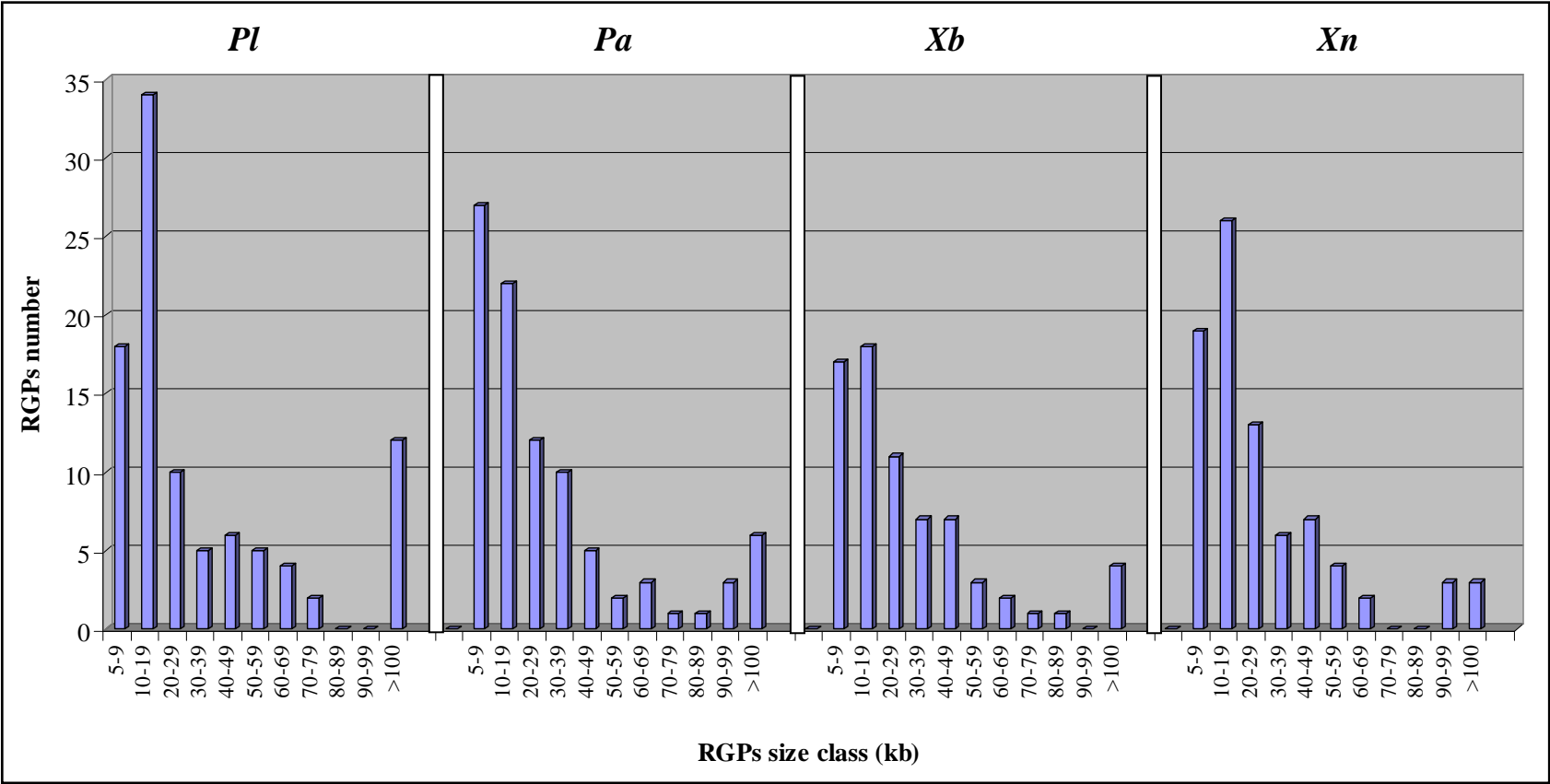

Supplement: Additional File 3 — Distribution of RGP sizes in the Photorhabdus luminescens TT01 (Pl), Photorhabdus asymbiotica ATCC43949 (Pa), Xenorhabdus nematophila ATCC19061 (Xn) and Xenorhabdus bovienii SS-2004 genomes (Xb). A figure showing the distribution of RGP size. [file 1471-2164-11-568-S3.PDF]

Additional file 4

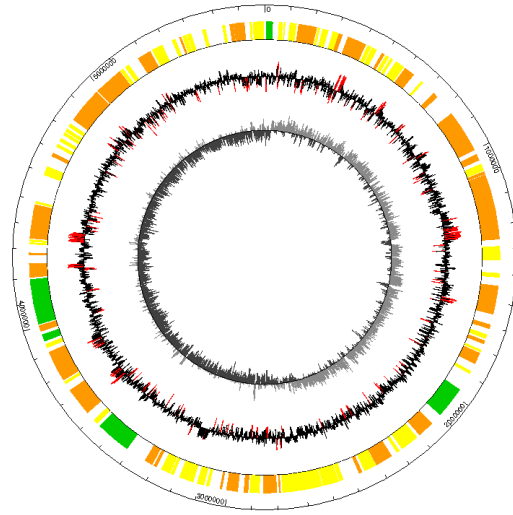

*P. luminescens*

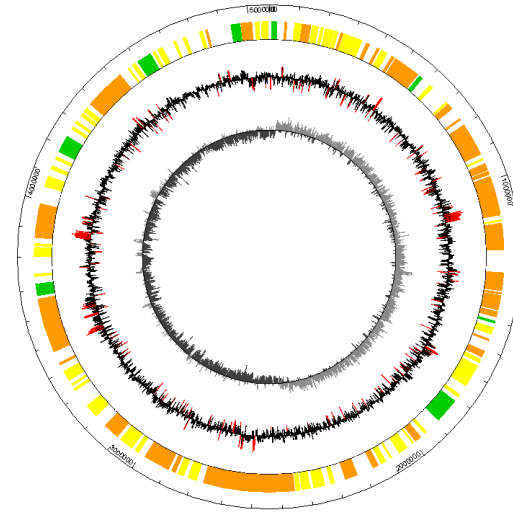

*P. asymbiotica*

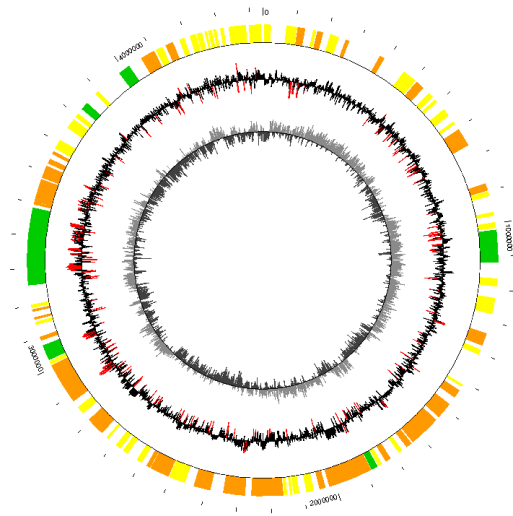

*X. nematophila*

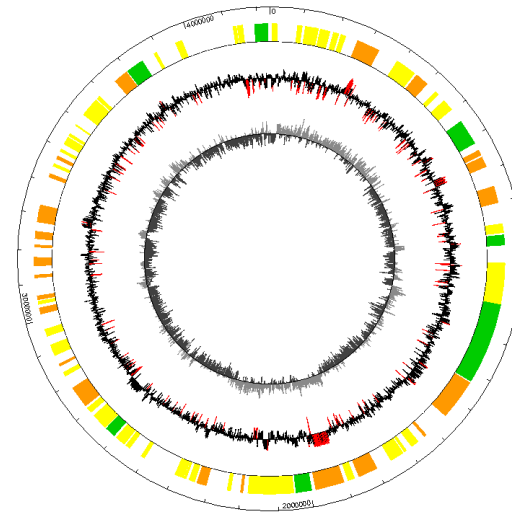

*X. bovienii*

Supplement: Additional File 4 — Schematic diagram of the distribution of RGPs sensu lato on the circular chromosomes of P. luminescens TT01 (Pl), P. asymbiotica ATCC43949 (Pa), X. nematophila ATCC19061 (Xn) and X. bovienii SS-2004 (Xb). Successive circles from inside to outside: GC skew; GC deviation (with values exceeding +/- 2 standard deviations indicated in red). Distribution of the different RGP types: GIs (orange), Phages (green) and RGPmob and RGPnone (yellow). A figure showing schematic diagrams of the distribution of RGPs. [file 1471-2164-11-568-S4.PDF]
