# Supplementary material for: Units of plasticity in bacterial genomes: new insight from the comparative genomics of two bacteria interacting with invertebrates, Photorhabdus and Xenorhabdus
Source: BMC Genomics. 2010 Oct 15;11:568. doi: 10.1186/1471-2164-11-568 (PMC3091717; doi:10.1186/1471-2164-11-568)
Supplement: Additional File 6 — Primers used in the study. A table listing the primers used in this study. [file 1471-2164-11-568-S6.DOC]

**Additional file 6:** Primers used in the study

| **RGP target** | **Primer** | **Sequence** | **Used for** |
| --- | --- | --- | --- |
| **locus D** | Plu1858F (P1) | **ATACCTGATGCAAGGGAACG** | **multiplex PCR & sequencing** |
|  | Plu1865R (P2) | **ACC TGC TCC ATT TTT CTG ACA T** | **multiplex PCR** |
|  | Plu1876F (P3) | **TCT GTT ACC TTA CCG GCC TAA A** | **multiplex PCR** |
|  | Plu1877Rbis (P4) | **TAT CCG GGC CTT ACT CCA AT** | **multiplex PCR & sequencing** |
|  | Plu1870F | **ATCCACCATCCGAACAAATGT** | **sequencing** |
|  | Plu1871F | **TCGTTCATCGACTCAAGTGC** | **sequencing** |
|  | Trp_F | **GAT GAC GCT GGC GGT ACC** | **sequencing** |
|  | Primer5_F | **ACT GTT TCA CCT TCG CCT TG** | **sequencing** |
|  | Trp_R | **AAT TAC ACC TGG GTT GTC ATC A** | **sequencing** |
| **locus E** | Plu2187F (P1) | **GAT GAG AAT GGT GGA TCG AGA** | **multiplex PCR & sequencing** |
|  | Plu2191R (P2) | **TACGGGCTTGTAGATCATTGG** | **multiplex PCR** |
|  | Plu2200F (P3) | **ATTCCTACAACCCCAGAACCTT** | **multiplex PCR** |
|  | Plu2201R (P4) | **AAC TTA CTC GGT GTG GGA TCT G** | **multiplex PCR & sequencing** |
|  | Plu2190 | **AGGATCGATGGCTTGATGAC** | **sequencing** |
|  | Plu2190Fbis | **ATT CCT CCG GTA ATA ATC TGT C** | **sequencing** |
|  | Plu2194R | **GCCCGTTTCAGCAATGTACT** | **sequencing** |
| **locus F** | Plu2467F (P1) | **CCG GTA TAG AAA GAC GAG GTG** | **multiplex PCR & sequencing** |
|  | Plu2468R (P2) | **CAA CTC TCT CTC CAA TGG CTA** | **multiplex PCR** |
|  | Plu2476F (P3) | **GAA TAT GCA GCT ACT GGG GGT A** | **multiplex PCR** |
|  | Plu2478R (P4) | **ACAGGCACTGCAACAGTAAGAC** | **multiplex PCR & sequencing** |
| **locus I** | Plu3378F (P1) | **AAG CGG GTC TCT CGT TAC AA** | **multiplex PCR & sequencing** |
|  | Plu3382R (P2) | **GTT GGC CGG GGA TAT TAG TT** | **multiplex PCR** |
|  | Plu3487Fbis (P3) | **CGGTGGCAAGATATCGACTT** | **multiplex PCR** |
|  | Plu3488R (P4) | **CGG AGT GGG AGA AAT CAC AA** | **multiplex PCR & sequencing** |
|  | ITS_3487,3488_F | **TTCCGTCTGGTTCATCACTG** | **sequencing** |
